# Listeria monocytogenes Bacteremia in an Individual With AIDS: A Case Report

**DOI:** 10.7759/cureus.95308

**Published:** 2025-10-24

**Authors:** Amar Patel, Julie Machen

**Affiliations:** 1 Internal Medicine, Cone Health, Greensboro, USA

**Keywords:** bacteremia, gram-postive rod, hiv, immunocompromised, listeria monocytogenes

## Abstract

*Listeria monocytogenes* is a gram-positive bacterium commonly found in soil, water, and rotting vegetation. The most common presentation of a *Listeria *infection is gastroenteritis. Invasive infection and severe complications are more common in immunocompromised hosts. Early identification and treatment of listeriosisis essential to reduce morbidity and mortality. This is a case report of a 50-year-old man with poorly controlled human immunodeficiency virus who presented with vague symptoms of chills and malaise. The patient was admitted for suspected pneumonia, later found to have *L. monocytogenes *bacteremia. We described his evaluation, treatment, and clinical course. The patient was treated with IV ampicillin and discharged with trimethoprim-sulfamethoxazole.

## Introduction

*Listeria monocytogenes* is a gram-positive rod that is commonly found in decaying vegetation, soil, and water [1]. Common foods that can increase the risk of *L. monocytogenes* infections include cold deli meats, cold hot dogs, unpasteurized milk, and raw sprouts [2]. Infections with *L. monocytogenes* are generally associated with immunocompromised individuals or pregnant women [2]. In elderly patients and immunocompromised patients, the bacteria can invade the bloodstream, causing sepsis, meningitis, or endocarditis, which is often fatal [3]. This case report describes an atypical presentation of a *L. monocytogenes* infection in an immunocompromised host.

## Case presentation

History

A 50-year-old man with acquired immunodeficiency syndrome (AIDS), chronic obstructive pulmonary disease (COPD), previous *Pneumocystis jirovecii* (PJP) pneumonia, and a history of pulmonary tuberculosis presented to the emergency department with a one-day history of nonproductive cough, chills, nausea, and malaise. He denied fevers, recent travel, sick contacts, or night sweats. He denied eating raw or uncooked foods. The patient had no recent hospitalizations. On presentation, the patient was taking prednisone 20 milligrams for five days for dermatitis and was also on trimethoprim-sulfamethoxazole for PJP prophylaxis. The patient’s HIV treatment included darunavir-cobicistat-emtricitabine-tenofovir alafenamide and dolutegravir, to which he was adherent.

Physical exam

The vitals showed a temperature of 97.8 degrees Fahrenheit, a heart rate of 119 beats per minute, a blood pressure of 108/67, a respiratory rate of 20, with oxygen saturations at 94% on room air. On exam, the patient looked lethargic and had dry mucous membranes. He was also tachycardic with bibasilar rales on lung exam. During the exam, the patient was somnolent but arousable, protecting his airway.

Investigation

Labs revealed a negative respiratory viral panel (severe acute respiratory syndrome (SARS) coronavirus, influenza A, influenza B, and respiratory syncytial virus). The comprehensive metabolic panel showed abnormalities of creatinine at 1.61 mg/dL, potassium at 3.2 mmol/L, aspartate aminotransferase at 93 U/L, and alanine transaminase at 47 U/L (Table 1). The complete blood count showed a WBC count of 8.6 K/uL, hemoglobin of 13.6 g/dL, and platelet count of 154 K/uL. His HIV viral load was 43,100 copies/mL, and his CD4 count was 95 cells/uL. He tested negative for Legionellaand strep antigens.

**Table 1:** Laboratory test results

Laboratory test (Units)	Patient Value	Reference Range
White Blood Cell (K/uL)	8.6	4.0-10.5
Hemoglobin (g/dL)	13.6	13.0-17.0
Platelets (K/uL)	154	150-400
Creatinine (mg/dL)	1.61	0.61-1.24
Potassium (mmol/L)	3.2	3.5-5.1
Aspartate Aminotransferase (U/L)	93	15-41
Alanine Transaminase (U/L)	47	0-44
Respiratory Panel (COVID, Influenza A and B, Respiratory Syncytial Virus)	Negative	Negative
HIV Viral Load (Copies/mL)	43,100	Undetectable
CD4 Count (cells/uL)	95	400-1790
Legionella Pneumonia Antigen	Negative	Negative
*Streptococcus pneumoniae* Antigen	Negative	Negative
Blood Cultures	Listeria Monocytogenes	Negative

**Figure 1:**
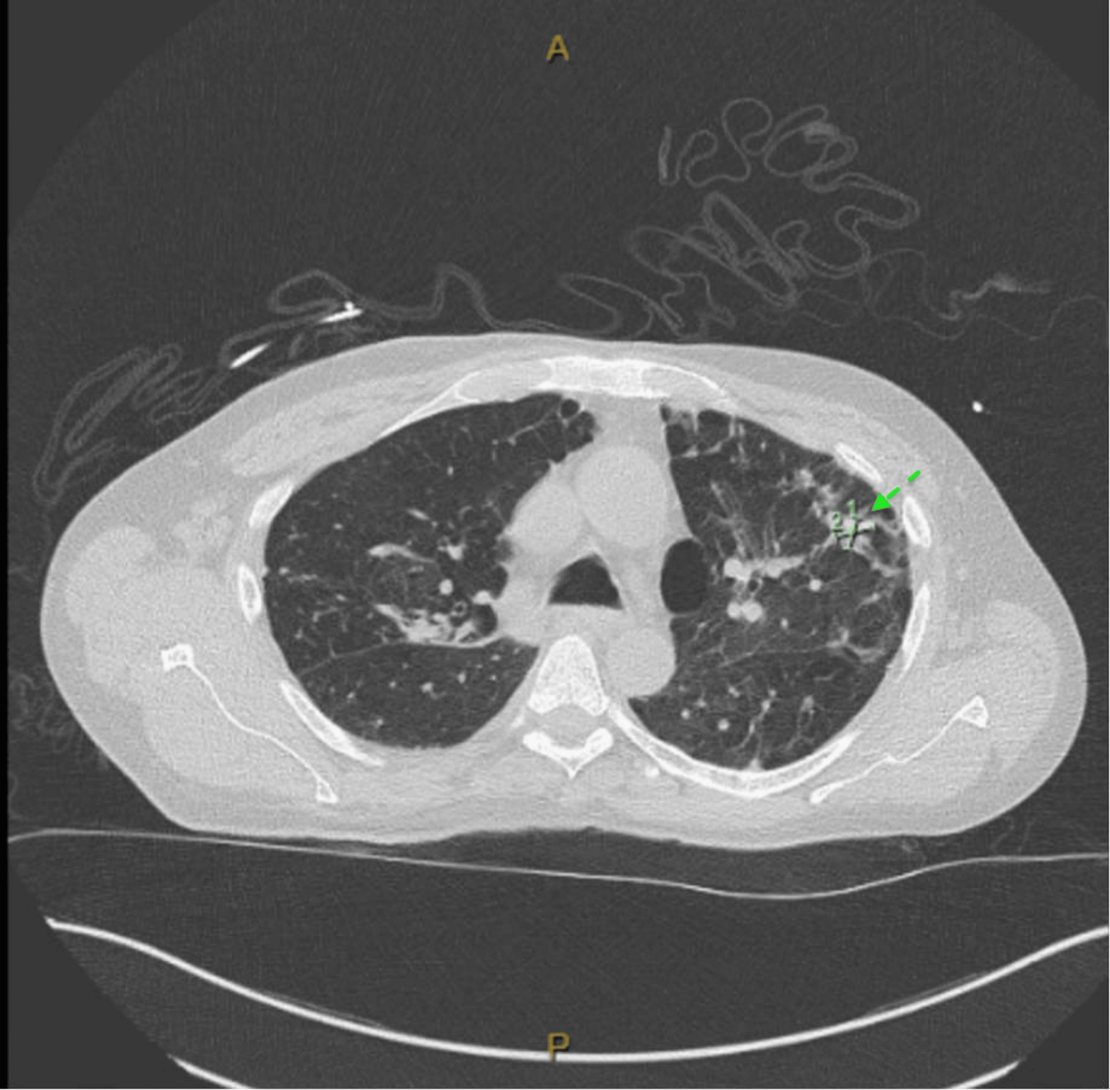
Chest CT showing evidence of tree-in-bud opacities concerning for pneumonia

**Figure 2:**
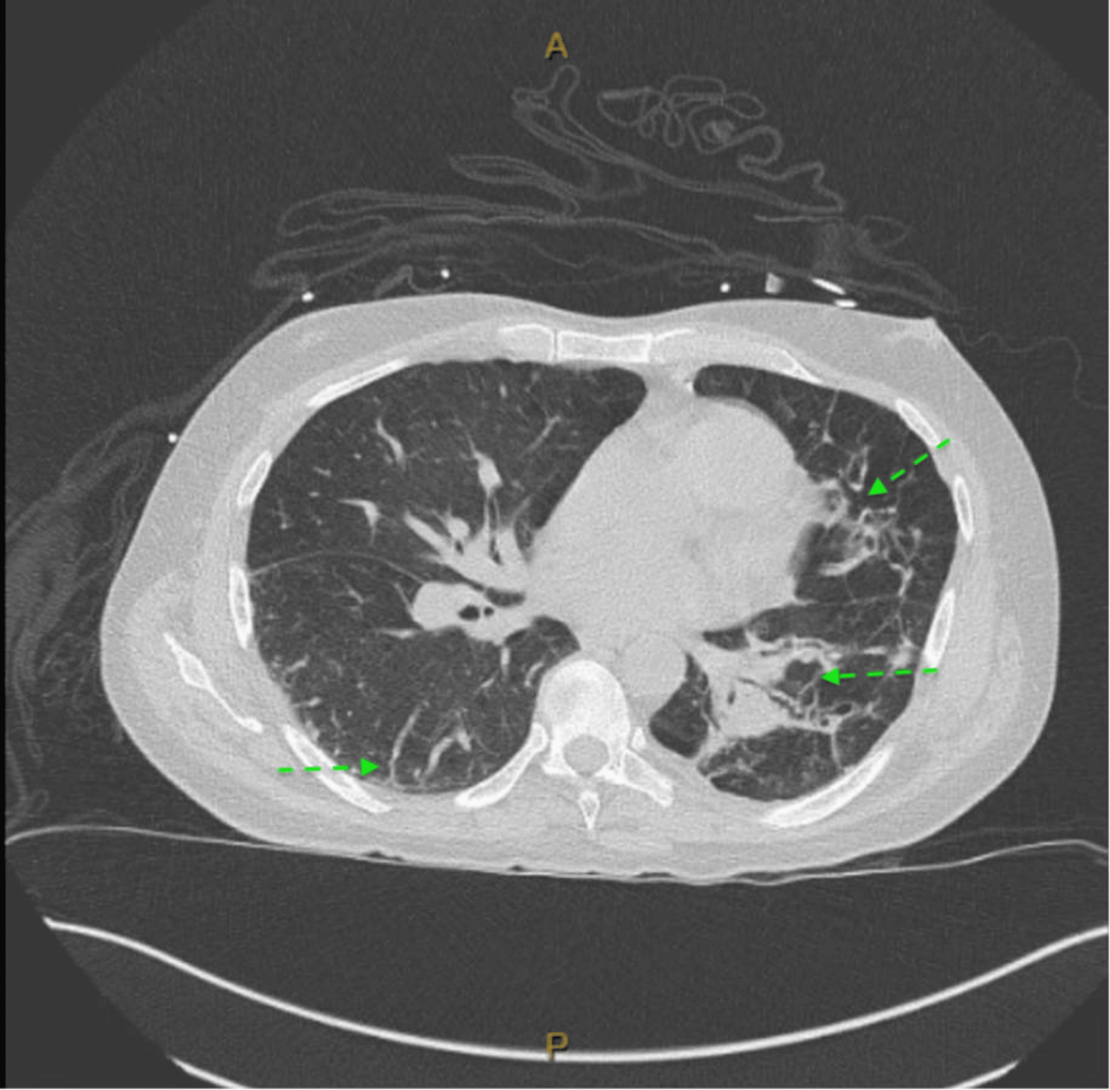
Chest CT showing evidence of tree-in-bud opacities concerning for pneumonia with background scarring and emphysema

Blood cultures were obtained from the patient upon admission. His chest X-ray showed possible trace pleural effusion and emphysema. With no obvious findings on the X-ray, a CT of the chest was ordered, which revealed new tree-in-bud opacities in the right lower lung lobe as well as emphysematous changes (Figures 1, 2).

Clinical course

The patient was admitted for pneumonia and started on cefepime and azithromycin for empiric community-acquired pneumonia coverage in an immunocompromised adult. On day two of the hospitalization, blood cultures came back positive for *L. monocytogenes*, and the patient was transitioned to IV ampicillin. A transthoracic echocardiogram was obtained, and it did not show evidence of vegetations. A transesophageal echocardiogram was not pursued as the patient was clinically improving without any clinical signs of endocarditis. Repeat blood cultures, after initiation of antibiotics, showed no growth of *Listeria*. The patient improved and was discharged on a two-week course of double-strength trimethoprim-sulfamethoxazole. He was set up with close follow-up with Infectious Disease for HIV management. The patient followed up in the clinic three weeks after discharge, and CD4 count had dropped to 60, and HIV viral load had dropped to 268. He continues to follow up as an outpatient with his most recent CD4 count of 87 and HIV viral load of less than 20. 

## Discussion

*L. monocytogenes* infections rank as the third leading cause of mortality from foodborne illness in the United States [4]. There are approximately 1600 cases of *L. monocytogenes* reported in the United States annually [5]. Complications include invasive bacteremia, endocarditis, and meningitis [2]. The mortality rates for invasive *L. monocytogenes* infections are high. The case fatality rate for *Listeria *bacteremia alone is estimated to be 34% [6]. With central nervous system involvement, the case fatality rate can be up to 27% [7]. The mortality rates increase for patients with* L. monocytogenes* infective endocarditis, ranging between 37% and 50% [8]. In this case, the patient did have bacteremia, but no signs of central nervous system involvement. Therefore, a lumbar puncture was not pursued. The incidence of infective endocarditis in patients with L. monocytogenes infection is rare, approximately 13-25/100,000 persons per year [8]. However, given this patient's risk factors and positive blood cultures, a transthoracic echocardiogram (TTE) was performed, ruling out endocarditis. A transesophageal echocardiogram was considered; however, given clinical improvement, negative repeat blood cultures, and normal TTE, this was deferred.

It is important to recognize and treat the infection from *Listeria *as delay in care can increase the all-cause mortality in patients with *L. monocytogenes *bacteremia. In one study, inadequate empiric treatments have been associated with significantly higher 30-day mortality rates [9]. The appropriate narrowed treatment for someone with *L. monocytogenes* infection includes amoxicillin, ampicillin, or aminoglycoside [10]. As soon as our patient was found to have* L. monocytogenes*, the antibiotics were narrowed to target the causative organism. 

The patient presented with vague symptoms, which made the differential broad. The imaging findings gave insight into the patient’s symptoms. The patient met the criteria for sepsis as he had end-organ damage (acute kidney injury with elevated creatinine) along with a source of infection (*L. monocytogenes* bacteremia) [11]. With the thought of pneumonia, IV cefepime and azithromycin were started for community-acquired pneumonia coverage in an immunocompromised patient. The patient was already on prophylaxis for PJP with trimethoprim-sulfamethoxazole. According to current practices, an antipseudomonal drug is indicated for patients who are immunocompromised; in some cases, where patients are not improving despite appropriate treatment, broadening coverage, or adding antifungals such as fluconazole would be the next step in the treatment [12]. In this case, the patient was improving with the treatment that was started; therefore, antifungal coverage was deferred. The surprising aspect of this course was the positive blood culture result of *Listeria.* The other interesting aspect of this case is the fact that the patient was already on trimethoprim-sulfamethoxazole, but still developed a *Listeria *infection. This brought up the question of adherence. With his uncontrolled HIV status, we felt that the patient may not have been taking his medications as instructed, playing a role in his presentation. 

For this patient, the source of the infection was not identified. *L. monocytogenes* is commonly found in the soil, groundwater, and animal feces [12]. It is also found in deli meats [13]. The patient denied exposure to these risk factors. Given his uncontrolled HIV status, he was at a higher risk of developing such an infection. According to the National Institute of Health, patients with HIV should avoid eating foods such as raw eggs, deli meats, undercooked meats, and unpasteurized products to reduce the risk of developing a foodborne illness [14]. This patient had not been counseled on foods to avoid prior to this hospitalization. Although we were unable to identify the source of the infection, we were able to counsel the patient on risk factors regarding diet. 

## Conclusions

*Listeria *infections can present in different ways. We presented a case of a *Listeria* infection in an AIDS patient. Vigilance is required for atypical presentations due to this organism. If the infection goes unrecognized, it can lead to severe outcomes. In this case, the patient had uncontrolled HIV, which made the patient more susceptible to developing such an aggressive infection. The patient was treated with the appropriate care and made a full recovery. The patient was counseled on a proper diet for his immunocompromised state to reduce future risk of infection.
